# Locoregionally recurrent head and neck squamous cell carcinoma: incidence, survival, prognostic factors, and treatment outcomes

**DOI:** 10.18632/oncotarget.16340

**Published:** 2017-03-17

**Authors:** Jer-Hwa Chang, Chia-Che Wu, Kevin Sheng-Po Yuan, Alexander T.H. Wu, Szu-Yuan Wu

**Affiliations:** ^1^ Division of Pulmonary Medicine, Department of Internal Medicine, Wan Fang Hospital, Taipei Medical University, Taipei, Taiwan; ^2^ School of Respiratory Therapy, College of Medicine, Taipei Medical University, Taipei, Taiwan; ^3^ Department of Otorhinolaryngology, Wan Fang Hospital, Taipei Medical University, Taipei, Taiwan; ^4^ The Ph.D. Program for Translational Medicine, Taipei Medical University, Taipei, Taiwan; ^5^ Institute of Toxicology, College of Medicine, National Taiwan University, Taipei, Taiwan; ^6^ Department of Radiation Oncology, Wan Fang Hospital, Taipei Medical University, Taipei, Taiwan; ^7^ Department of Internal Medicine, School of Medicine, College of Medicine, Taipei Medical University, Taipei, Taiwan

**Keywords:** head and neck cancer, recurrence, incidence, survival, prognostic factors

## Abstract

**Purpose:**

For locoregionally recurrent head and neck squamous cell carcinoma (HNSCC), appropriate therapeutic decisions remain unclear. We examined the treatment outcomes of a national cohort to determine suitable treatments for and prognostic factors in patients with locoregionally recurrent HNSCCs at different stages and sites.

**Patients and methods:**

We analyzed data of >20-year-old patients with HNSCC at American Joint Committee on Cancer clinical stages I–IV without metastasis from Taiwan National Health Insurance and cancer registry databases. The index date was the date of recurrent HNSCC diagnosis. Recurrent HNSCC was defined as the annotation of locoregional recurrence with tissue proof in cancer registry databases. The enrolled patients were categorized into three groups: Group 1 comprised those undergoing chemotherapy (CT) alone; Group 2 comprised those receiving reirradiation (re-RT) alone (total radiation dose ≥ 60 Gy through intensity modulation radiation therapy [IMRT]); Group 3 comprised those receiving concurrent chemoradiotherapy (CCRT) alone (irradiation total dose ≥60 Gy through IMRT); and Group 4 comprised those receiving salvage surgery with or without RT or CT.

**Results:**

We enrolled 4,839 and 28,664 HNSCC patients with and without locoregional recurrence, respectively (median follow-up, 3.25 years). Locoregional recurrence rate and incidence were 14.44% and 40.73 per 1,000 person-years, respectively. Age ≥ 65 years, Charlson comorbidity index (CCI) score > 6, advanced clinical stage at first diagnosis, and recurrence-free interval < 1 year were significant independent prognostic risk factors for overall survival as per univariate and multivariate Cox regression analyses. After adjusting for age, sex, CCI scores, clinical stage at first diagnosis, and recurrence-free interval, adjusted hazard ratios (aHRs; 95% confidence intervals [CIs]) for overall mortality in recurrent clinical stages I and II were 0.63 (0.45–0.89, *p* = 0.009), 0.65 (0.52–0.83, *p* < 0.001), and 0.32 (0.26–0.40, *p* < 0.001) in Groups 2, 3, and 4, respectively, whereas they were 1.23 (0.99–1.52, *p* = 0.062), 0.69 (0.60–0.79, *p* < 0.001), and 0.39 (0.34–0.44, *p* < 0.001) for Groups 2, 3, and 4, respectively, for overall mortality in recurrent clinical stage III and IV.

**Conclusions:**

Age, CCI score, clinical stage at first diagnosis, and recurrence-free interval are significant independent prognostic factors for overall survival of recurrent HNSCC patients. Regardless of recurrence stage or site, salvage surgery is the recommended first recurrent HNSCC treatment choice. Re-RT alone and CCRT are more suitable for inoperable recurrent early-stage oral and nonoral cavity recurrent HNSCCs, respectively.

## INTRODUCTION

Treatments for locoregionally recurrent head and neck squamous cell carcinoma (HNSCC) including combined modality approaches—salvage surgery, reirradiation (re-RT), and chemotherapy (CT) as well as concurrent chemoradiotherapy (CCRT)—generally ensure long-term control.[1–4] Recurrent HNSCC is a major cause of morbidity, preventing long-term survival of HNSCC patients. Locoregional recurrences are noted in 15%-50% of patients with HNSCC, a major factor contributing toward head and neck cancer-related deaths.[5–7] Recurrent HNSCC is difficult to treat for multiple reasons; these include the effects of prior treatment on tumor cells and the infiltrative and the multifocal nature of HNSCC, a typical characteristic of recurrent disease in this area.[8]

Most studies have analyzed recurrent metastatic secondary HNSCCs, primary HNSCCs, and new head and neck tumors [1, 9–11] by using a heterogenous population.[11–15] Because patients with a second primary malignancy may have more favorable prognoses than do those with true recurrences, recurrent cancers should be distinguished from second primary malignancies.[14, 15] Some studies included relatively heterogeneous recurrent head and neck cancer to evaluate a single treatment modality; however, the pathological types of the cancer considered were heterogenous (e.g., nasopharyngeal cancer and salivary gland cancer), not squamous cell carcinoma alone.[3] Laryngeal and nasopharyngeal cancers have a more favorable prognosis than do those at other sites.[3]

Salvage surgery may be associated with a range of postoperative complications,[4, 16] and the benefits of re-RT are uncertain.[14, 17, 18] Tortochaux et al.[18] reported that palliative-intent re-RT of approximately 50 Gy for vague recurrent clinical stage and secondary primary HNSCCs was not beneficial. Intensity modulation radiation therapy (IMRT) at a high radiation dose (≥60 Gy) has shown more favorable overall survival in heterogeneous populations containing patients with nasopharyngeal cancer, salivary gland cancer, laryngeal cancer, metastatic tumors, multifocal cancer sites, unclear recurrence stage, recurrent HNSCC, and secondary primary cancer.[3, 19, 20]

To resolve these complicated questions, in this study, we enrolled patients with only locoregionally recurrent HNSCC. CT alone may be selected in cases of multiple comorbidity, old age, advanced recurrence stage, and difficult-to-approach cancer site. However, the benefits of other treatment approaches (e.g., re-RT, CCRT, and salvage surgery) and the optimal therapeutic decisions for patients with recurrent HNSCC or prognostic factors remain unclear. In this study, we explored the treatment outcomes of a national cohort from Taiwan to determine the optimal treatment strategy for improving the likelihood of survival in patients with different recurrent cancer stages and sites. Furthermore, we explored the clinical prognostic factors of recurrent HNSCC patients.

## PATIENTS AND METHODS

In this study, two cohorts were created using data from the Taiwan National Health Insurance (NHI) and cancer registry databases, both of which cover approximately 99% of the entire population of Taiwan. We enrolled patients diagnosed with HNSCC during January 1, 2002-December 31, 2011. The follow-up duration was from the index date to December 31, 2013. The research-oriented data sets, released by Taiwan NHI Administration through the Collaboration Center of Health Information Application (CCHIA), include all original claims data and registration files of beneficiaries enrolled in the NHI program. Thus, the CCHIA data can be used for tracing all medical services used by all patients with HNSCC in Taiwan. The cancer registry database of the CCHIA contains abundant cancer-related information, including the clinical stage, treatment modalities, pathology, RT doses, RT techniques, and regimens used—CT, CCRT, or sequential CT and RT.[21] Before accessing the data sets, researchers must sign an agreement to protect patient privacy, after which researchers can access the CCHIA database only for analyzing specific topics. Patient identification numbers in the data sets are encrypted, which prevents specific patient identification.[22] Here, the diagnoses of enrolled patients were confirmed according to their pathological data, and patients with new or recurrent HNSCC diagnoses were confirmed to have no other cancer or distant metastasis. The inclusion criteria were HNSCC (identified according to International Classification of Diseases, Ninth Revision, Clinical Modification [ICD-9-CM] codes 140.0-148.9), age > 20 years, and American Joint Committee on Cancer clinical cancer stages I-IV without metastasis. The exclusion criteria were a history of cancer before HNSCC diagnosis, distant metastasis, missing sex data, age < 20 years, nasopharyngeal cancer, laryngeal cancer, in situ carcinoma, sarcoma, salivary gland cancer, and HNSCC recurrence. The index date was the date of diagnosis of recurrent HNSCC. Recurrent HNSCC was defined as the annotation of locoregional recurrence with pathological proof in cancer registry databases. We also excluded patients with recurrent HNSCC who did not receive any treatments, did not receive RT after first HNSCC diagnosis, did not use re-RT through IMRT, received a re-RT dose of <60 Gy, or received re-RT with stereotactic body RT. Here, the CT regimen included cisplatin, docetaxel, gemcitabine, 5-fluorouracil, hydroxyurea, methotrexate, carboplatin, and paclitaxel; however, platinum-based CT was administered in most recurrent HNSCC patients as per the Taiwan NHI policy. Finally, we enrolled HNSCC patients with and without locoregional recurrence and categorized them into the following groups on the basis of the treatment modality to compare their outcomes: Group 1, those undergoing CT alone; Group 2, those undergoing re-RT alone (irradiation total dose ≥ 60 Gy through IMRT); Group 3, those receiving CCRT (irradiation total dose ≥ 60 Gy through IMRT); and Group 4, those receiving salvage surgery with or without RT or CT. Comorbidity was scored using the Charlson comorbidity index (CCI).[23] Only comorbidities observed 6 months before and after the index date were included: the comorbid conditions were identified and included according to the main ICD-9-CM diagnosis code for the first admission or more than two repeated main diagnosis codes for visits to the outpatient department. Significant independent predictors such as age, sex, CCI score, clinical stage at first diagnosis, and recurrence-free interval were determined using a multivariate Cox regression analysis to determine the hazard ratio (HR); the independent predictors were controlled for or stratified in the analysis, and the endpoint was the mortality rate among treatments, with Group 1 as the control arm.

The cumulative incidence of death was estimated using the Kaplan–Meier method, and the differences among treatment modalities were determined using the log-rank test. After adjusting for confounders, the Cox proportional method was used to model the time from the index date to all-cause mortality among patients undergoing the treatments. In the multivariate analysis, HRs were adjusted for age, sex, CCI, clinical stage at first diagnosis, and recurrence-free interval. Stratified analyses were performed to evaluate the mortality risk associated with different treatment modalities and with salvage surgery or nonsurgical intervention among treatments for different recurrent cancer stages and sites (oral and nonoral cavity). All analyses were performed using SAS (version 9.3; SAS, Cary, NC, USA). A two-tailed *p* value of <0.05 was considered statistically significant.

## RESULTS

We enrolled 28,664 HNSCC patients without locoregional recurrence and 4,839 recurrent HNSCC patients without distant metastasis (Table 1), both with a median follow-up duration after the index date of 3.15 (interquartile range, 2.55) years. Incidence for locoregional recurrence was 40.73 per 1,000 person-years (PY) and the locoregional recurrence rate was 14.44%. More than 60% of recurrent HNSCC patients were at the advanced clinical stage at first diagnosis (stages III and IV) and more than 85% were of working age, mostly younger than 65 years. Recurrence rates in the oral cavity, oropharynx, and hypopharynx were 15.45%, 11.05%, and 9.90%, respectively. Groups 1, 2, 3, and 4 comprised 680, 208, 904, and 2,247 patients, respectively (Table 2). In all four groups, re-RT alone was selected for a higher proportion of elderly patients (age ≥ 65 years, 31.25%; mean age in Group 2, 56.96 years); by contrast, a higher proportion of younger patients (age < 65 years) underwent CCRT or surgery with or without RT or CT (90.71% vs. 86.78% of the patients). The most predominant recurrence site was the oral cavity—572 (84.12%), 181 (87.12%), 801 (88.60%), and 1,940 (78.80%) patients in Groups 1, 2, 3, and 4, respectively. The clinical stages at first diagnosis differed in all four groups; a higher proportion of patients at clinical stage IV underwent CT alone (57.06% of stage IV patients in Group 1), whereas a lower proportion of these patients underwent surgery with or without RT or CT (31.60% of stage IV patients in Group 4). The recurrence-free interval was less than 1 year and less than 2 years in >60% and >85% of the patients, respectively. Advanced HNSCC stage at first diagnosis was associated with a higher recurrence rate (Table 2). The mortality rates were 75%, 76.44%, 71.79%, and 56.21% in Groups 1, 2, 3, and 4, respectively. The mortality rates per 100 PY were 75.10, 65.16, 56.70, and 22.31 in Groups 1, 2, 3, and 4, respectively.

**Table 1:** Characteristics of HNSCC patients with and without locoregional recurrence

Locoregional recurrence status	Nonlocoregionally recurrent HNSCC patients (*N* = 28664)		Locoregionally recurrent HNSCC patients (*N* = 4839)		
Variable	*n*	(%)	*n*	(%)	*p* value*
Sex					<0.001
Male	26174	(85.29)	4516	(14.71)	
Female	2490	(88.52)	323	(11.48)	
Age (years)					<0.001
20–35	1107	(83.61)	217	(16.39)	
36–49	10661	(83.72)	2073	(16.28)	
50–64	11856	(86.03)	1925	(13.97)	
≥65	5040	(88.98)	624	(11.02)	
Cancer site					<0.001
Oral cavity	22685	(84.55)	4145	(15.45)	
Oropharynx	2583	(88.95)	321	(11.05)	
Hypopharynx	3393	(90.10)	373	(9.90)	

*p values were calculated using a chi-square test.

Row percentages are presented in this table.

**Table 2:** Characteristics of recurrent HNSCC patients treated with different treatment modalities

Treatment group	CT alone (1) (*n* = 680)		Re-RT alone (2) (*n* = 208)		CCRT (3) (*n* = 904)		Surgery ± RT/CT (4) (*n* = 2247)	
Variable	*n*	(%)	*n*	(%)	*n*	(%)	*n*	(%)
Sex								
Male	646	(95.00)	184	(88.46)	846	(93.77)	2107	(93.77)
Female	34	(5.00)	24	(11.54)	58	(6.42)	140	(6.23)
Age (Mean [SD], years)	52.06	(10.83)	56.96	(13.59)	49.64	(10.10)	51.52	(10.66)
Age (years)								
20–34	21	(3.09)	7	(3.37)	39	(4.31)	68	(3.03)
35–49	251	(36.91)	53	(25.48)	393	(43.47)	884	(39.34)
50–64	305	(44.85)	83	(39.90)	388	(42.92)	998	(44.41)
≥65	103	(15.15)	65	(31.25)	84	(9.29)	297	(13.22)
Clinical stage at first diagnosed								
I	54	(7.94)	31	(14.90)	144	(15.93)	538	(23.94)
II	114	(16.76)	47	(22.60)	194	(21.46)	583	(25.95)
III	124	(18.24)	39	(18.75)	172	(19.03)	416	(18.51)
IV	388	(57.06)	91	(43.75)	394	(43.58)	710	(31.60)
Recurrence site								
Oral cavity	572	(84.12)	181	(87.02)	801	(88.60)	1940	(86.34)
Oropharynx	54	(7.94)	14	(6.73)	49	(5.42)	153	(6.81)
Hypopharynx	54	(7.94)	13	(6.25)	54	(5.97)	154	(6.85)
Recurrence-free interval								
3–6 months	146	(21.47)	67	(32.21)	284	(31.42)	651	(28.97)
7–12 months	314	(46.18)	74	(35.58)	325	(35.95)	936	(41.66)
1–2 years	110	(16.18)	24	(11.54)	107	(11.84)	294	(13.08)
2–3 years	37	(5.44)	12	(5.77)	51	(5.64)	124	(5.52)
3–5 years	42	(6.18)	17	(8.17)	82	(9.07)	159	(7.08)
>5 years	31	(4.56)	14	(6.73)	55	(6.08)	83	(3.69)
CCI score								
0	174	(25.59)	37	(17.79)	188	(20.80)	478	(21.27)
1–5	175	(25.74)	56	(26.92)	162	(17.92)	428	(19.04)
6–9	310	(45.59)	105	(50.48)	531	(58.74)	1284	(57.14)
≥10	21	(3.09)	10	(4.81)	23	(2.54)	57	(2.54)
No. of deaths	512	(75.00)	159	(76.44)	649	(71.79)	1263	(56.21)
Mortality rate per 100 PY	75.10		65.16		56.70		22.31	

Abbreviations: RT, radiotherapy; CT, chemotherapy; CCRT, concurrent chemoradiotherapy; CCI, Charlson comorbidity index; PY, person-years; SD, standard deviation.

Age, sex, CCI score, clinical stage at first diagnosis, and recurrence-free interval were significant independent predictors according to multivariate Cox regression analysis (Table 3). Age ≥ 65 years, CCI score > 6, advanced clinical stage at first diagnosis, and recurrence-free interval < 1 year were significant independent prognostic risk factors for overall survival identified in both univariate and multivariate Cox regression analyses (Table 3). Recurrence duration from cancer diagnosed > 1 year, CCRT, and surgery with or without RT or CT were significant independent prognostic protective factors of overall survival (denoted by HRs and 95% confidence intervals [CIs]) were 0.69 (0.63-0.76, *p* < 0.001) for recurrence-free interval of >1 year, 0.70 (0.62-0.79, *p* < 0.001) for CCRT, and 0.37 (0.34-0.42, *p* < 0.001) for surgery with or without RT or CT in both univariate and multivariate Cox regression analyses (Table 3).

**Table 3:** Cox regression analysis for the risk of death among recurrent HNSCC patients

	Univariate Analysis			Multivariate Analysis*		
Variable	HR	(95% CI)	*p* value	HR	(95% CI)	*p* value
Treatment group (Reference group: CT alone)						
Re-RT alone (2)	1.02	(0.86–1.22)	0.817	1.00	(0.83–1.20)	0.992
CCRT (3)	0.72	(0.64–0.81)	<0.001	0.70	(0.62–0.79)	<0.001
Surgery ± RT/CT (4)	0.37	(0.33–0.41)	<0.001	0.37	(0.34–0.42)	<.0001
Age ≥ 65 years	1.11	(1.00–1.24)	0.058	1.13	(1.01–1.26)	0.040
Men	1.09	(0.93–1.28)	0.299	1.10	(0.93–1.29)	0.263
CCI score > 6	1.08	(1.07–1.10)	<0.001	1.09	(1.07–1.10)	<.0001
Clinical stage at first diagnosis	1.31	(1.27–1.36)	<0.001	1.24	(1.20–1.29)	<.0001
Recurrence-free interval > 1 year	0.65	(0.59–0.71)	<0.001	0.69	(0.63–0.76)	<.0001

*All above variables were used in multivariate analysis.

Abbreviations: RT, radiotherapy; CT, chemotherapy; CCRT, concurrent chemoradiotherapy; CCI, Charlson comorbidity index; CI, confidence interval; HR, hazard ratio.

Stratified analysis was performed to evaluate the mortality risk among treatment modalities for different recurrent cancer stages (stages I and II as well as III and IV) and sites (oral and nonoral cavity; Tables 4 and 5). A stratified Cox proportional hazard model was used to analyze the risk of death and the associated treatment modality among recurrent HNSCC patients (Table 4). Groups 2, 3, and 4 functioned as the control arm (Group 1) for investigating the mortality risk after treatments. After adjustment for age, sex, CCI score, clinical stage at first diagnosis, and recurrence-free interval, adjusted HRs (aHRs; 95% CIs) for overall mortality in recurrent clinical stages I and II were 0.63 (0.45-0.89, *p* = 0.009), 0.65 (0.52-0.83, *p* < 0.001), and 0.32 (0.26-0.40, *p* < 0.001) in Groups 2, 3, and 4, respectively (Table 4), whereas those for overall mortality in recurrent clinical stages III and IV were 1.23 (0.99-1.52, *p* = 0.062), 0.69 (0.60-0.79, *p* < 0.001), and 0.39 (0.34-0.44, *p* < 0.001) for in Groups 2, 3, and 4, respectively. Another stratified analysis was performed to evaluate the mortality risk among treatment modalities for patients with oral and nonoral cavity HNSCCs: among patients with recurrent oral cavity HNSCCs, aHRs (95% CIs) for overall mortality were 1.01 (0.83-1.23, *p* = 0.952), 0.73 (0.64-0.83, *p* < 0.001), and 0.38 (0.34-0.43, *p* < 0.001) in Groups 2, 3, and 4, respectively, whereas among those with recurrent nonoral cavity HNSCCs, aHRs were 0.97 (0.59-1.58, *p* = 0.891), 0.52 (0.39-0.71, *p* < 0.001), and 0.33 (0.25-0.43, *p* < 0.001) in Groups 2, 3, and 4, respectively (Table 4). Considering both recurrent clinical stages and sites, the stratified Cox proportional hazard model for the risk of death and the associated treatment modality among recurrent HNSCC patients were analyzed (Table 5). This stratified analysis was performed to evaluate the mortality risk among treatment modalities for oral and nonoral cavity HNSCC patients with recurrent clinical stages I and II or III and IV; among patients with recurrent oral cavity HNSCCs with recurrent early stages, aHRs (95% CIs) for overall mortality were 0.62 (0.43-0.89, *p* = 0.009), 0.68 (0.53-0.86, *p* = 0.002), and 0.32 (0.26-0.41, *p* < 0.001) in Groups 2, 3, and 4, respectively, whereas they were 1.28 (1.01-1.62, *p* = 0.041), 0.72 (0.62-0.84, *p* < 0.001), and 0.40 (0.35-0.46, *p* < 0.001) in Groups 2, 3, and 4, respectively, among those with recurrent oral cavity HNSCCs with recurrent advanced stage (Table 5). Among patients with recurrent nonoral cavity HNSCCs at recurrent early stages, aHRs (95% CIs) for overall death were 0.90 (0.26-3.06, *p* = 0.859), 0.48 (0.17-1.43, *p* = 0.188), and 0.26 (0.10-0.63, *p* = 0.003) in Groups 2, 3, and 4, respectively, whereas they were 0.98 (0.56-1.69, *p* = 0.928), 0.53 (0.38-0.73, *p* < 0.001), and 0.33 (0.25-0.43, *p* < 0.001) in Groups 2, 3, and 4, respectively, among those with recurrent nonoral cavity HNSCCs with recurrent advanced stage (Table 5)

**Table 4:** Stratified Cox proportional hazard model for the risk of death and the associated treatment modalities among recurrent HNSCC patients

Stratified Variables	Treatment modality	*n*	No. of deaths (%)	aHR* (95% CI)	*n* value
Recurrent clinical stage					
Stage I and II	CT alone	168	110 (65.48)	1.00	
Stage I and II	Re-RT alone	78	50 (64.10)	0.63 (0.45–0.89)	0.009
Stage I and II	CCRT	338	209 (61.83)	0.65 (0.52–0.83)	<0.001
Stage I and II	Surgery ± RT/CT	1121	541 (48.26)	0.32 (0.26–0.40)	<0.001
Stage III and IV	CT alone	512	400 (78.13)	1.00	
Stage III and IV	Re-RT alone	130	109 (83.85)	1.23 (0.99–1.52)	0.061
Stage III and IV	CCRT	566	440 (77.74)	0.69 (0.60–0.79)	<0.001
Stage III and IV	Surgery ± RT/CT	1126	722 (64.12)	0.39 (0.34–0.44)	<0.001
Recurrent Cancer site					
Oral cavity	CT alone	572	421 (73.60)	1.00	
Oral cavity	Re-RT alone	180	137 (76.11)	1.01 (0.83–1.23)	0.952
Oral cavity	CCRT	797	572 (71.77)	0.73 (0.64–0.83)	<0.001
Oral cavity	Surgery ± RT/CT	1932	1072 (55.49)	0.38 (0.34–0.43)	<0.001
Nonoral cavity	CT alone	108	89 (82.41)	1.00	
Nonoral cavity	Re-RT alone	28	22 (78.57)	0.97 (0.59–1.58)	0.891
Nonoral cavity	CCRT	107	77 (71.96)	0.52 (0.39–0.71)	<0.001
Nonoral cavity	Surgery ± RT/CT	315	191 (60.63)	0.33 (0.25–0.43)	<0.001

*HRs were adjusted by age, sex, CCI score, clinical stage at first diagnosis, and recurrence-free interval

Abbreviations: CT, chemotherapy; CCRT, concurrent chemoradiotherapy; CCI, Charlson comorbidity index; CI, confidence interval; aHR, adjusted hazard ratio; PY, person-years; RT, radiotherapy.

**Table 5:** Stratified Cox proportional hazard model for the risk of death and the associated treatment modalities among recurrent HNSCC patients, considering both recurrent clinical stages and sites

Recurrence stage	Recurrent site	*n*	Treatment modality	aHR (95% CI)	*n* value
Group I					
Stage I and II	Nonoral cavity	8	CT alone	1.00	
Stage I and II	Nonoral cavity	7	Re-RT alone	0.90 (0.26–3.06)	0.859
Stage I and II	Nonoral cavity	13	CCRT	0.48 (0.17–1.43)	0.188
Stage I and II	Nonoral cavity	64	Surgery ± RT/CT	0.26 (0.10–0.63)	0.003
Group II					
Stage I and II	Oral cavity	160	CT alone	1.00	
Stage I and II	Oral cavity	71	Re-RT alone	0.62 (0.43–0.89)	0.009
Stage I and II	Oral cavity	325	CCRT	0.68 (0.53–0.86)	0.002
Stage I and II	Oral cavity	1057	Surgery ± RT/CT	0.32 (0.26–0.41)	<0.001
Group III					
Stage III and IV	Nonoral cavity	100	CT alone	1.00	
Stage III and IV	Nonoral cavity	21	Re-RT alone	0.98 (0.56–1.69)	0.928
Stage III and IV	Nonoral cavity	94	CCRT	0.53 (0.38–0.73)	<0.001
Stage III and IV	Nonoral cavity	251	Surgery ± RT/CT	0.33 (0.25–0.43)	<0.001
Group IV					
Stage III and IV	Oral cavity	412	CT alone	1.00	
Stage III and IV	Oral cavity	109	Re-RT alone	1.28 (1.01–1.62)	0.041
Stage III and IV	Oral cavity	472	CCRT	0.72 (0.62–0.84)	<0.001
Stage III and IV	Oral cavity	875	Surgery ± RT/CT	0.40 (0.35–0.46)	<0.001

*HRs were adjusted by age, sex, CCI score, clinical stage at first diagnosis, and recurrence-free interval

Abbreviations: CT, chemotherapy; CCRT, concurrent chemoradiotherapy; CCI, Charlson comorbidity index; CI, confidence interval; aHR, adjusted hazard ratio; PY, person-years; RT, radiotherapy.

**Figure 1:**
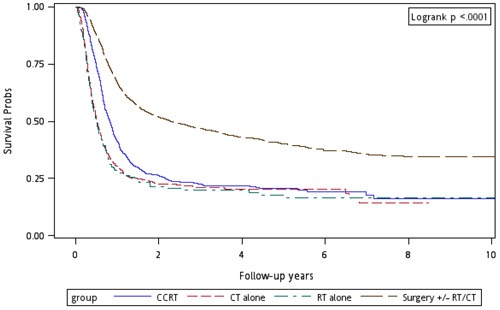
Kaplan–Meier curves for overall survival of patients undergoing different treatments

**Figure 2:**
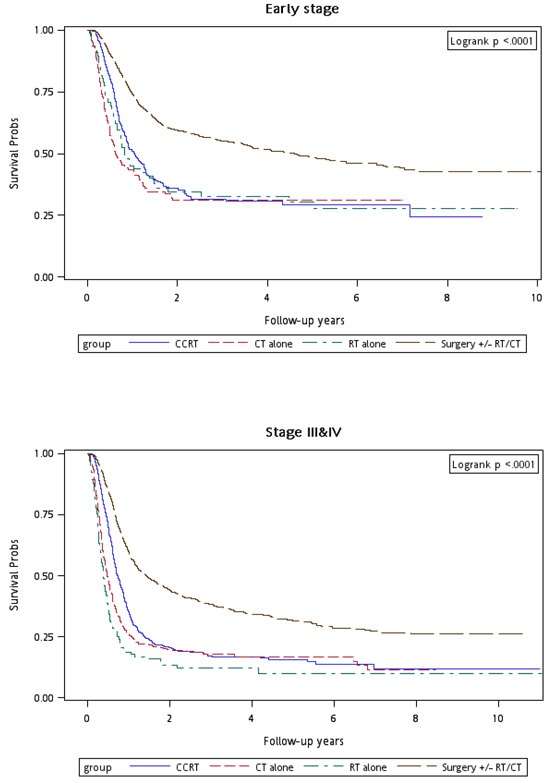
Kaplan–Meier curves for overall survival of patients undergoing different treatments and stratified by different AJCC clinical cancer stages

**Figure 3:**
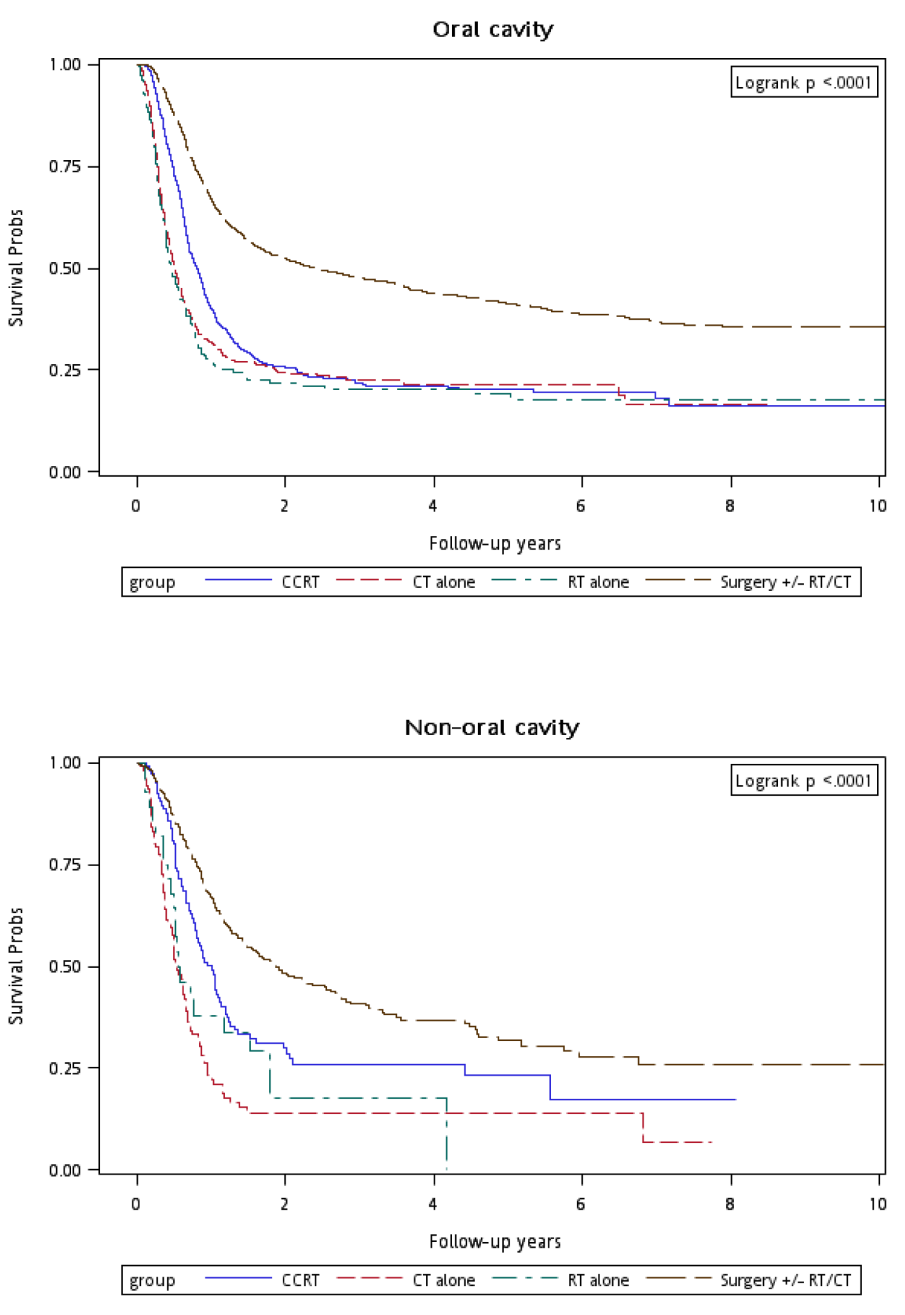
Kaplan-Meier curves for overall survival of patients undergoing different treatments and stratified by oral cavity or non-oral cavity cancers

Figure 1 shows the Kaplan–Meier overall survival curves of patients in the four treatment arms. The highest overall survival rate was noted in Group 4 patients (log-rank test, *p* < 0.0001). The 5-year overall survival rates were 22.53%, 19.44%, 22.71%, and 46.74% in Groups 1, 2, 3, and 4, respectively. Figure 2 shows the Kaplan–Meier overall survival curves of patients in the four treatment arms with early and advanced recurrent clinical stages. The survival rates of Group 4 patients were higher than those of Group 1, 2, and 3 patients at the same recurrent clinical stage (log-rank test, *p* < 0.0001): the 5-year overall survival rates were 31.94% (CT alone) 31.52% (re-RT alone), 30.53% (CCRT), and 54.95% (surgery ± RT/CT) at early stages, whereas they were 21.13% (CT alone), 13.34% (re-RT alone), 20.09% (CCRT), and 37.93% (surgery ± RT/CT) at advanced stages, respectively. Figure 3 shows the Kaplan–Meier overall survival curves of patients undergoing different treatments for oral or nonoral cavity HNSCCs. In Group 4, surgery with or without RT or CT resulted in a higher overall survival of recurrent HNSCC patients with oral or nonoral cavity HNSCCs (log-rank test, *p* < 0.0001). The 5-year overall survival rates were 23.44% (CT alone), 21.62% (re-RT alone), 22.53% (CCRT), and 46.75% (surgery ± RT/CT) for oral cavity HNSCCs, whereas they were 13.63% (CT alone), 0% (re-RT alone), 25.19% (CCRT), and 36.95% (surgery ± RT/CT) for nonoral cavity HNSCCs (Figure 3).

## DISCUSSION

This is the first study that specifically investigated recurrent HNSCCs, excluding secondary primary HNSCC, metastatic HNSCC, nasopharyngeal cancer, laryngeal cancer, or salivary gland cancer. The homogeneity of our population was more suitable for evaluating different treatments to determine the optimal therapeutic approach for recurrent HNSCC. In addition, we stratified recurrent HNSCCs into different recurrent clinical stages and sites. Most HNSCC cases in Taiwan were oral cavity HNSCCs; this aided in obtaining abundant data regarding the effects of different treatment modalities for recurrent oral or nonoral cavity HNSCCs in Taiwan and in determining potential predictors of recurrent HNSCC and differences in the outcomes of recurrent oral and nonoral cavity HNSCCs. Numerous HNSCC cases have been noted in Taiwan; therefore, this study also facilitated the determination of the prognostic factors for recurrent HNSCC (Table 3).

In Taiwan, more than 99% of head and neck cancer cases are HNSCCs, and more than 88% of head and neck cancer patients have a habit of betel nut chewing.[24, 25] Betel nut chewers show higher incidence of locoregional recurrence and secondary primary cancers as well as poorer disease-specific and overall survival than do nonchewers.[24] Liao et al.[24] considered the incidence of locoregional recurrence as well as that of secondary primary cancers, whereas we considered the incidence of only locoregional recurrence and not secondary primary cancers. This study is the first to report the incidence for locoregional recurrence alone (40.73 per 1,000 PY) as well as the locoregional recurrence rate (14.44%) in areas with high proportions of betel nut chewers. The recurrence rates in the oral cavity, oropharynx, and hypopharynx were 15.45%, 11.05% and 9.90%, respectively. Advanced HNSCC stage at first diagnosis was associated with higher recurrence rate (Table 2). These findings are similar to those of Brockstein et al., who showed that locoregional recurrence is rare in patients with early-stage tumors at first diagnosis.[6] Consistent with our results that poor overall survival is associated with recurrent clinical stage, Goldstein et al.[26] and Mendenhall et al.[26] showed that extensive recurrent disease is associated with less favorable outcomes.

Salama et al.[2] and Spencer et al.[14] reported that a longer recurrence-free interval affords a more favorable prognosis. Our multivariate Cox regression analysis for the risk of death among recurrent HNSCC patients showed that HR of overall death was 0.69 when the recurrence-free interval was longer than 1 year. Tanvetyanon et al.[28] showed that medical comorbidity has a considerable negative effect on survival. In this study, age ≥ 65 years, CCI score > 6, advanced clinical stage at first diagnosis, and recurrence-free interval < 1 year were significant independent prognostic risk factors for overall survival of recurrent HNSCC patients. In addition, recurrence-free interval > 1 year, CCRT, and surgery with or without RT or CT were significant independent protective prognostic factors for overall survival. This is the first study to report the prognostic factors for survival after different treatments for recurrent HNSCC.

In our study, more than 60% and 85% of patients had recurrence-free intervals of <1 and <2 years, respectively (Table 2). The stage of recurrent HNSCC affects surgical outcomes, which induce operative mortality and significant complications.[4, 16] These phenomena indicate the importance of early detection of recurrence (within 2 years after first HNSCC diagnosis post treatments) because poor overall survival was associated with recurrent clinical stage. In addition, early-stage HNSCCs were easier to operate with more favorable overall survival observed in our data (Tables 2 and 3). The 5-year overall survival rate was 54.95% for recurrent early-stage HNSCC in Group 4 (Figure 2).

In our study, age at recurrence was less than 65 years in more than 85% of the patients; recurrence at a younger age can cause a significant negative economic impact on patients’ families.[26] Cancer in working-age patients affects the patients’ families, care providers, and has various effects on the economy. Because incidence of locoregional recurrence is higher in the working-age population, determining an optimal therapeutic modality is crucial. A stratified analysis was performed to evaluate the mortality risk among treatment modalities for different recurrent cancer stages and sites. Our results showed that re-RT alone and CCRT were significantly superior to CT alone for recurrent early-stage HNSCC, with similar aHRs of 0.63 and 0.65, respectively. De Crevoisier et al. also reported similar outcomes of CCRT and re-RT alone.[13] According to our results, the optimal therapeutic approach for recurrent HNSCC was surgery with or without RT or CT, with the lowest aHR at 0.32. The potential possibility of superior survival in group 4 might be selected patients who are candidates for definitive therapy but have unresectable locally recurrent diseases in group 1, 2, and 3. However, our results and the trend of therapeutic outcomes were similar to those of previous studies.[1, 3, 11, 19] In this study, we suggested that recurrent early-stage HNSCC should be treated with salvage surgery with or without RT or CT as first choice. If the patient is inoperable, re-RT alone or CCRT can be feasibly applied for recurrent early-stage HNSCC. Furthermore, according to the results, salvage surgery or CCRT is significantly superior to CT alone, but not re-RT alone. We suggest salvage surgery for recurrence stage III and IV HNSCC if the patient is operable; however, if patient is inoperable, CCRT is recommended rather than re-RT alone.

We performed another stratified analysis for evaluating the mortality risk among treatment modalities for patients with oral and nonoral cavity HNSCCs. This is the first study to estimate the outcomes of different treatments for recurrent oral or nonoral cavity HNSCCs. Our results showed that salvage surgery and CCRT are significantly superior to CT alone, but not for re-RT alone, regardless of the recurrence site. In terms of mortality risk reduction, CCRT was more effective in reducing mortality risk among nonoral cavity HNSCC patients (aHR, 0.52) than among oral cavity HNSCC patients (aHR, 0.73), suggesting that CCRT is more effective against recurrent nonoral cavity HNSCCs than against recurrent oral cavity HNSCCs. Similar to its effect on new HNSCC,[27] the therapeutic effect of CCRT on recurrent nonoral cavity HNSCC is more favorable than on recurrent oral cavity HNSCC.

Considering both recurrent clinical stages and sites, we evaluated a stratified Cox proportional hazard model for the risk of death and the associated treatment modality among recurrent HNSCC patients (Table 5). This stratified analysis was performed to evaluate the mortality risk for treatment modalities administered to patients with oral and nonoral cavity HNSCCs at recurrent clinical stage I and II or III and IV. For recurrence stage I and II oral cavity HNSCC, re-RT alone was significantly superior to CT alone but similar to CCRT. Salvage surgery was the most favorable choice for operable recurrence stage I and II oral cavity HNNSCC patients. For recurrence stage III and IV oral cavity HNSCC, re-RT alone was significantly inferior to CT alone and CCRT and salvage surgery were significantly superior to CT alone. Based on our results, we recommend surgery as the first choice for treating recurrent oral cavity HNSCC, regardless of the recurrence stage. If the patient is inoperable, re-RT alone or CCRT may be feasible for treating recurrence stage I and II oral cavity HNSCCs, whereas CCRT may be suitable for recurrence stage III and IV oral cavity HNSCCs rather than re-RT alone.

Our findings for recurrent early-stage nonoral cavity HNSCC differed from those for oral cavity HNSCCs. Re-RT alone was not significantly superior to CT alone, even for recurrence stage I and II nonoral cavity HNSCCs. By contrast, CCRT and salvage surgery were significantly superior to CT alone, regardless of the recurrence stage. Thus, we recommend surgery as the first choice for recurrent nonoral cavity HNSCCs, regardless of the recurrence stage. CCRT may be suitable for inoperable recurrent nonoral cavity HNSCCs at any stage rather than re-RT alone.

Here, patients with recurrent HNSCC undergoing re-RT alone had the oldest mean age (56.96 years). By contrast, those undergoing CT alone were relatively young compared with those undergoing re-RT alone. In our study, age ≥ 65 years, CCI score > 6, advanced clinical stage at first diagnosis, and recurrence-free interval < 1 year were significant independent prognostic risk factors of overall survival in both univariate and multivariate Cox regression analyses. After adjustment for the aforementioned risk factors, re-RT alone was applicable only to inoperable patients with recurrent early-stage oral cavity HNSCCs. By contrast, CCRT was suitable for all inoperable HNSCCs, regardless of the location or stage. Our study further increases the uncertainty of the benefits of re-RT.[14, 17, 18] Our findings may aid clinicians in selecting treatment modalities specific for our recurrent HNSCC types.

The strength of this study is the large sample size and homogeneity of the population for recurrent HNSCC. The results suggest that aggressive treatments (e.g., surgery, CCRT, and re-RT alone) can reduce the incidence of death in patients with selected recurrent HNSCC. This is the first study indicating the optimal therapeutic decisions for patients with recurrent HNSCC according to the recurrent cancer sites and stages: aggressive treatments are more suitable; this should be considered in future clinical studies. However, this study has limitations. First, the toxicity induced by aggressive treatments could not be determined; therefore, the treatment-related mortality estimates may have been biased. Second, information regarding the human papillomavirus (HPV) test is not recorded in the databases used in this study; hence, the effect of different treatments on HPV-positive and -negative patients could not be examined. Third, all investigated patients with HNSCC were from an Asian population, and ethnic susceptibility was unclear; hence, our results should be cautiously extrapolated to non-Asian populations. Fourth, the relatively small number of patients with recurrence stage I and II nonoral cavity HNSCCs might limit the generalizability of our conclusions; thus, a large-scale randomized trial in which carefully selected patients undergoing suitable aggressive treatments and palliative or supportive care approaches are used for comparison is essential for obtaining crucial information regarding population specificity and disease occurrence. Fifth, diagnoses of all comorbid conditions were completely dependent on ICD-9-CM codes; nevertheless, the Taiwan NHI Administration randomly reviews charts and interviews patients to verify the accuracy of the diagnoses, and hospitals with outlier chargers or practices may undergo an audit and subsequently receive heavy penalties if malpractice or discrepancies are identified. Sixth, to prevent creating several subgroups, the various procedures of salvage surgery and CT regimen were not categorized separately during analyses; the effects of different CT regimens and surgical procedures are unclear. Seventh, most oncologists In Taiwan follow the indications for postoperative reirradiation were margin positive or lymph nodes with extracapsular extension based on NCCN guidelines and Janot's study [1, 28]. However, the real pathologic risk features could be missing in the current data. Finally, the NHI database contains no information on tobacco use, alcohol consumption, dietary habits, socioeconomic status, or body mass index, all of which may be mortality risk factors. Nevertheless, given the magnitude and statistical significance of the observed effects in this study, these limitations are unlikely to affect the conclusions.

## CONCLUSIONS

Age ≥ 65 years, CCI score > 6, advanced clinical stage at first diagnosis, and recurrence-free interval < 1 year were significant independent prognostic risk factors for overall survival of recurrent HNSCC patients. CCRT and surgery with or without RT or CT were significant independent prognostic protective factors for overall survival of recurrent HNSCC patients. Salvage surgery was the first choice for recurrent HNSCC, regardless of the recurrence stage or site. Re-RT alone may be suitable only for inoperable recurrent early-stage oral cavity HNSCCs. Although CCRT is suitable for patients with recurrent HNSCCs of any type, its therapeutic effect is more favorable in patients with recurrent nonoral cavity HNSCCs than in those with recurrent oral cavity HNSCCs.

## Abbreviations

RT: radiotherapy
CT: chemotherapy
CCHIA: Collaboration Center of Health Information Application
NHI: National Health Insurance
ICD: International Classification of Diseases
CCRT: concurrent chemoradiotherapy
HR: hazard ratio
CI: confidence interval
CCI: Charlson comorbidity index
HNSCC: head and neck squamous cell carcinoma
